# iSimp in BioC standard format: enhancing the interoperability of a sentence simplification system

**DOI:** 10.1093/database/bau038

**Published:** 2014-05-21

**Authors:** Yifan Peng, Catalina O. Tudor, Manabu Torii, Cathy H. Wu, K. Vijay-Shanker

**Affiliations:** ^1^Department of Computer and Information Sciences, University of Delaware, 18 Amstel Ave, Newark, DE 19716, USA and ^2^Center for Bioinformatics and Computational Biology, University of Delaware, 15 Innovation Way, Newark, DE 19711, USA

## Abstract

This article reports the use of the BioC standard format in our sentence simplification system, iSimp, and demonstrates its general utility. iSimp is designed to simplify complex sentences commonly found in the biomedical text, and has been shown to improve existing text mining applications that rely on the analysis of sentence structures. By adopting the BioC format, we aim to make iSimp readily interoperable with other applications in the biomedical domain. To examine the utility of iSimp in BioC, we implemented a rule-based relation extraction system that uses iSimp as a preprocessing module and BioC for data exchange. Evaluation on the training corpus of BioNLP-ST 2011 GENIA Event Extraction (GE) task showed that iSimp sentence simplification improved the recall by 3.2% without reducing precision. The iSimp simplification-annotated corpora, both our previously used corpus and the GE corpus in the current study, have been converted into the BioC format and made publicly available at the project’s Web site: http://research.bioinformatics.udel.edu/isimp/.

**Database URL:**http://research.bioinformatics.udel.edu/isimp/

## Introduction

With the accelerating growth of biomedical publications, biologists have difficulty in keeping up with the new findings reported in the papers. Natural language processing (NLP) techniques have thus been developed to process the biomedical texts. However, the syntactic complexity of the language poses a challenge in designing and applying NLP systems. One solution is to simplify sentences before applying NLP techniques, thus concealing the syntactic complexity from further NLP steps. For this purpose, we have previously developed iSimp (1), a sentence simplification system.

iSimp can be used as a preprocessing module to provide simplified text to enhance the performance of NLP systems and text mining (TM) applications. To integrate iSimp into wide-ranging applications, we need to design customized adapters for data exchange. Recently, the BioC format has emerged as a community standard for the exchange of text documents and annotations (2). Based on an XML format, BioC is simple, yet robust, and very suited for iSimp’s need.

We participated in the BioCreative IV Track 1 (BioC: Interoperability) and adopted the BioC format in iSimp. In this article, we report how BioC is used with iSimp, and how iSimp can be integrated with various applications. Overall, this work makes three main contributions.

The first contribution is the development of a BioC tag set for annotating simplification constructs. The tag set can be used in conjunction with any sentence simplification system to exchange data with other NLP systems. The standard tag set also serves the purpose of comparing the results among different simplification systems.

The second contribution is a mechanism of using the BioC framework. The proposed mechanism denotes simplified sentences in a corpus file, along with the annotation of simplification constructs in the original sentence. It allows simplified sentences to be included in the BioC annotation file so that they can be processed in place of (or in addition to) the original text. Moreover, the annotated phrases within simplified sentences can be mapped back to the original text. This mechanism is important for two reasons. First, it ensures that the output is presented aligned with the original text. Second, it allows the benchmarking of the NLP procedure, where the outputs must be aligned with the gold standard annotation in the original corpus.

The third contribution of this work is the construction of an iSimp corpus presented in the BioC format. The corpus, consisting of 130 Medline abstracts annotated with six types of simplification constructs, can be used for the evaluation of the simplifier. In addition to this corpus, we also transform the GENIA Event Extraction (GE) corpora of the BioNLP-ST 2011 to BioC format. The GE corpora were used to evaluate the impact of iSimp in relation extraction (RE) tasks. All these corpora have been made publicly available for evaluating and comparing various simplification systems.

To show the wide applicability and good performance of iSimp, we examined its impact on the RE task. We developed a basic rule-based RE system to recognize the BioC format, presented how iSimp could enhance its performance and showed that iSimp was seamlessly added to the RE system with little effort required for the system integration.

## Background

This section introduces the concepts and related work for sentence simplification and the BioC framework.

### Sentence simplification

Sentence simplification is a technique to detect various types of clauses and constructs contributing to the complexity of sentences, and to produce two or more simple sentences while maintaining both coherence and the communicated message. By reducing the complexity, sentence simplification can ease the development of NLP/TM tools, as well as other tools, such as machine translation tools. To illustrate the usefulness of sentence simplification, consider the following complex sentence from the biomedical literature:

E1. A third genetic linkage to disease is alpha-synuclein, a protein that is heavily phosphorylated in Lewy bodies and Lewy neuritis, the pathological hallmarks of PD. (PMID-22342821)

In this example, we can see coordination (e.g. ‘Lewy bodies and Lewy neuritis’), relative clause (e.g. ‘that is heavily phosphorylated in …’ referring to ‘a protein’) and apposition (e.g. ‘a protein that is …’ referring to ‘alpha-synuclein’ and ‘the pathological hall marks of PD’ referring to ‘Lewy bodies and Lewy neuritis’). These are major syntactic constructs that contribute to the complexity of sentences. After identifying these constructs, the complex sentence can be broken into multiple simple sentences. Here we show only two examples, which require combining the coordination with the relative clause and one of the appositions:

E2. Alpha-synuclein is heavily phosphorylated in Lewy bodies.

E3. Alpha-synuclein is heavily phosphorylated in Lewy neuritis.

Pattern-based or machine-learning approaches will undoubtedly find E2 and E3 much easier to process for the extraction of information/features than the original sentence.

The automatic simplification of sentences was first introduced by (3) to improve the performance of systems that rely on natural language input. It has been subsequently used in the biomedical domain. As a preprocessing module, syntactic simplification can be used to prune irrelevant constructs from shallow parsing results (4), parse trees (5), dependency graphs (6), or to produce different versions of the original sentence by splitting it in multiple sentences or by combining constituents (7–9). Also, some constructs, like coordination and apposition, can be assembled before pattern matching and unfolded after extraction (4, 7).

### BioC

BioC is a framework that aims to provide an easy and powerful way of integrating TM tools (2). It uses an XML format, which enables the sharing of documents and annotations (e.g. part-of-speech tags, named entities and entity relations). In the BioCreative IV workshop, many NLP tools incorporated the BioC format. They perform tasks such as abbreviations, semantic role labeling and gene normalization (10–13). The integration of iSimp with the BioC format is somewhat different from those cases because iSimp produces new sentences besides the tagging of the original text. These new sentences may include words that were not in the original text.

## Materials and Methods

In this section, we describe the methodology of iSimp and show how the BioC format is used to enhance the interoperability of iSimp.

### iSimp

**Figure 1.**
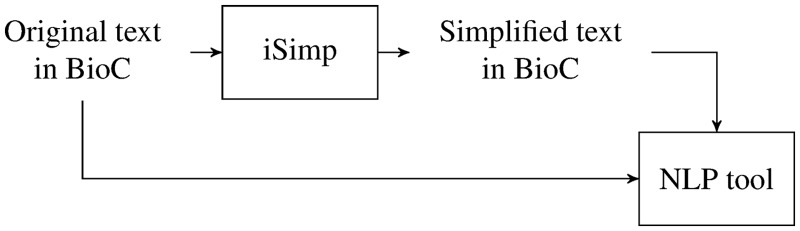
NLP pipeline with iSimp.

To make sentence simplification interoperable with other NLP/TM applications, we see a sentence simplifier as a module to be used at the beginning of NLP/TM pipelines. With this in mind, we designed and developed iSimp (http://research.bioinformatics.udel.edu/isimp). Figure 1 shows how iSimp can be used as a module in an NLP/TM pipeline. It is worth pointing out that iSimp is designed to act like an optional plug-in. This means other applications are not expected to make changes to use iSimp. Instead, we should be able to plug iSimp in/out as needed, where the application can access original sentences, simplified sentences or both.

Currently, iSimp can detect six types of simplification constructs: coordination, relative clause, apposition, introductory phrase, subordinate clause and parenthetical element. For a more detailed description of sentence simplification, as well as its challenges (e.g. attachment ambiguities, boundary detection and nested constructs), we refer the reader to (1).

**Figure 2.**
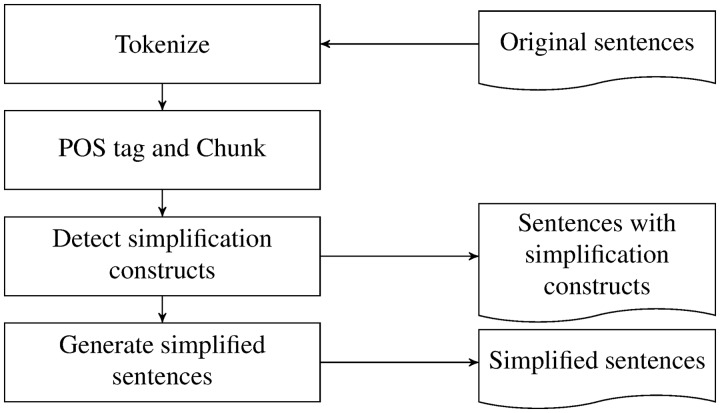
The workflow of iSimp.

In comparison with the works using parse trees or dependency graphs, iSimp uses shallow parsing and recursive transition networks to detect all forms of simplifications. Figure 2 shows the workflow of the system. iSimp first tokenizes the text, and then it splits each sentence into a sequence of nonoverlapping chunks. The detection of various simplification constructs is based on the chunks, and from these, iSimp generates simplified sentences. Three types of chunks were investigated here: noun phrases, verb groups and prepositional phrases.

iSimp scans the phrase sequence from left to right. Whenever a trigger word of a simplification construct is found (e.g. ‘and’ for coordination or ‘which’ for relative clause), we attempt to identify the simplification construct using transition networks. If a stop state of the network is found, then a simplification construct was detected successfully. We extended the network to address nested constructs. For an in-depth description of this process, we refer the reader to (1).

iSimp generates simplified sentences by combining various simplification constructs. To illustrate the problem, consider the following sentence:

E4. Active Raf-2 [_coordination_**phosphorylates** and activates] MEK1, [_relative clause_ which in turn [_coordination_**phosphorylates** and activates] the MAP kinases signal regulated kinases, [_coordination & appositive_ ERK1 and ERK2]]. (PMID-8557975)

iSimp is able to generate several simple sentences from (E4). Five of them are shown below:

E5. (a) Active Raf-2 **phosphorylates** MEK1.

   (b) MEK1 in turn **phosphorylates** ERK1.

   (c) MEK1 in turn **phosphorylates** ERK2.

   (d) The MAP kinases signal regulated kinases is an ERK1.

   (e) The MAP kinases signal regulated kinases is an ERK2.

Sometimes, iSimp will introduce new words in the simplified sentence to keep it grammatically correct. For example, in (E5d) and (E5e), we put ‘is an’ between the appositive clause and the singular noun phrase it refers to, to form a new sentence. Adding new words to the corpus is one of the factors that distinguish iSimp from other applications that enhance BioC.

### iSimp in BioC format

**Figure 3.**
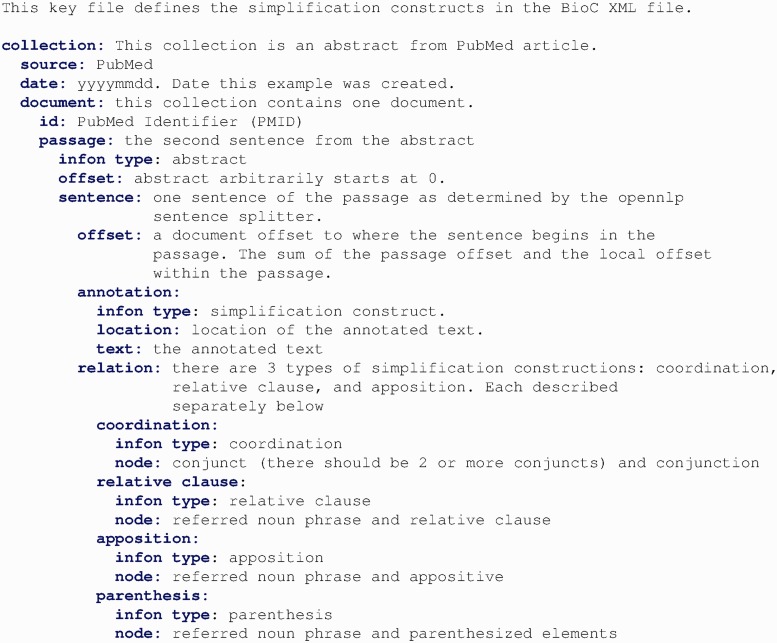
The key file used in iSimp to define the simplification constructs associated with the data.

**Figure 4.**
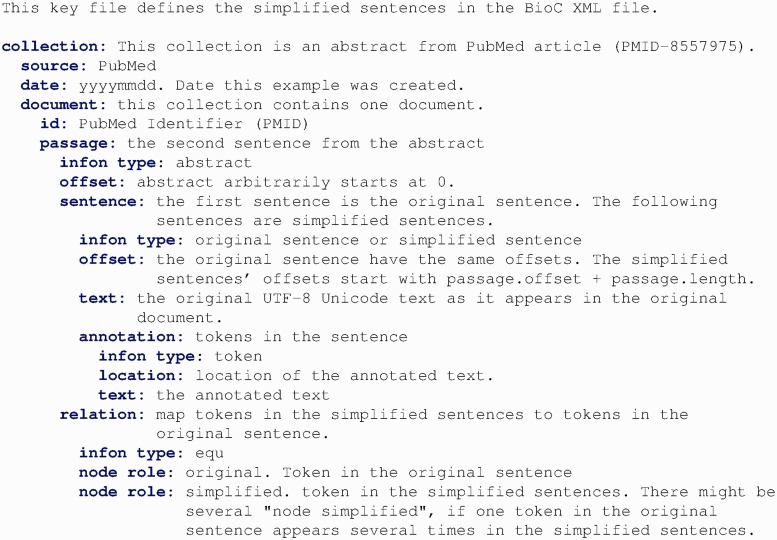
The key file used in iSimp to define the simplified sentences associated with the data.

Because sentence simplification requires a unique schema to add new text in the corpus, we designed a BioC tag set for annotating and sharing the simplification results. Figures 3 and 4 show the key file used in iSimp to define the semantics associated with the data.

**Figure 5.**
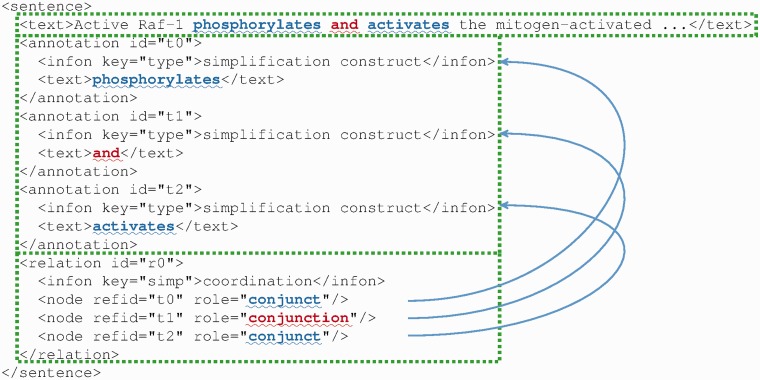
An example of sentence simplification annotation in BioC format. The coordination contains two conjuncts (‘phosphorylates’, ‘activates’) and one conjunction (‘and’). Some attributes, like the location elements, are not shown for the sake of space.

We use the annotation element to mark up the simplification construct components, and we use the relation element to specify how these components are related. In the latter, we further specify the name of the simplification type (e.g. coordination, relative clause, etc.), as well as roles for each component in the relation using the node element (e.g. ‘conjunct’ and ‘conjunction’ for the coordination, ‘referred noun phrase’ and ‘appositive’ for the apposition). For example, Figure 5 shows the coordination ‘phosphorylates and activates’ in BioC format. This coordination contains two conjuncts (‘phosphorylates’ and ‘activates’) and one conjunction (‘and’). Some attributes, like the location elements, are not shown in this figure for lack of space.

**Figure 6.**
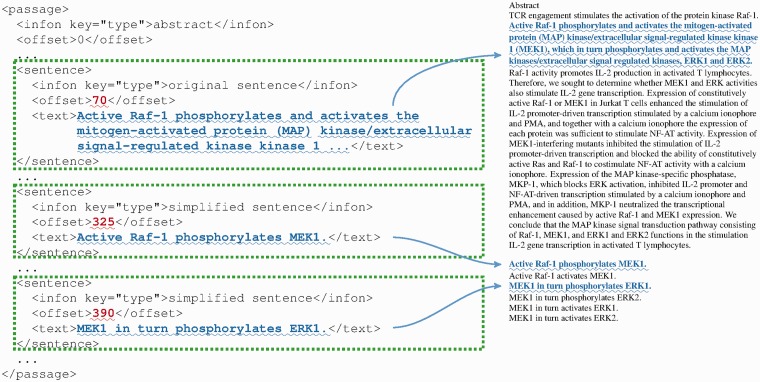
An example of simplified sentences in BioC format (left) and the corresponding text file (right) with locations highlighted.

As mentioned before, iSimp generates new simplified sentences. This poses an additional challenge to the integration of the BioC format, as such cases were not directly addressed in the original design of BioC (2). Hence, we designed and proposed a new way of using BioC framework. Figure 6 shows an example of simplified sentences in the BioC format (left), as well as the corresponding text file (right) with locations highlighted. As mentioned before, we include both original and simplified sentences in the BioC file. The offsets of the original sentences are the same as in the original text. However, the offsets of the simplified sentences start with the offset of the next character after the last character in the original document (offset of document + length of document). This new collection could then be treated as the input collection for the next step in the NLP pipeline.

**Figure 7.**
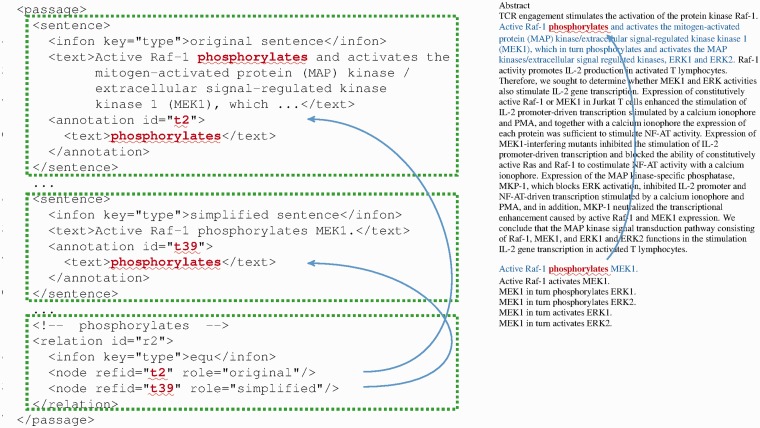
An example showing ‘equ’ (equivalence) relations in iSimp-generated BioC file.

To link text in simplified sentences to that in the original sentence, we introduce the ‘equ’ (equivalence) relation. Figure 7 shows an example of an equivalence relation, in which we link ‘phosphorylates’ back to the original sentence. This way phrases in the simplified sentences can be mapped back to the corresponding phrases in the original sentence. Equivalence relations can be used to ensure that downstream applications recognize the duplicated nature of such ‘equivalent’ phrases and do not report the same information multiple times in the end. Implementation of this mechanism was feasible owing to the extensibility of the BioC format.

### Online iSimp with BioC

For various NLP/TM applications to use sentence simplification, we have made iSimp available online. It adopts the BioC format and supports two interfaces.

Users can submit a document in the standard BioC format, which is described in Figure 5 of (2). The format requires a document to be specified as a sequence of sentences where the offsets are specified with respect to the whole document. Given the input file, iSimp will output the list of sentences marked with simplification constructions. Moreover, iSimp will append the simplified sentences to the marked input sentences, and provide this output as a zip file for download. For displaying different sentence simplification aspects, we have also developed a web interface where users can provide sentences in plain text and iSimp will output the sentences marked with simplification constructions directly in the browser.

To support interoperable machine-to-machine interaction with other applications, iSimp can be accessed by enclosing the BioC file in the POST requests. The iSimp Web server will accept and process one sentence per request and send back simplification constructs and simplified sentences in an all-in-one BioC file. This will guarantee the response time and avoid loading overly large BioC files. To submit sentences in one BioC file (BioC sentence Document type definition (DTD)), users can use the following format: http://research.bioinformatics.udel.edu/isimp/biocsentence?biocfile=BioCFileContent.

## Results and discussion

### Evaluation on RE system

To examine the usefulness of iSimp, we considered a very simple rule-based RE system. The first relation we focused on was the phosphorylation relation between the trigger and the theme (substrate) as defined in the GE corpora. We used straightforward rules, as shown below, where X is a noun phrase in which the protein or protein product appears as a headword:

1. phosphorylate (or, phosphorylates, phosphorylated, phosphorylating) X
2. phosphorylation of X
3. X phosphorylation
4. [_noun phrase_ phosphorylated X]

These rules are able to match straightforward mentions of phosphorylation in text. However, they will fail to find mentions of phosphorylation in complex sentences, like the one shown in (E4). However, the first rule can apply to the simplified (E5a)–(E5c) and extract <phosphorylates, MEK1>, <phosphorylates, ERK1> and <phosphorylates, ERK2>. As long as the rules for extraction are precise, the simplification step will help improve the recall of the system, without hurting the precision.

We evaluated iSimp in terms of the impact it had on the performance of the RE system. Thus, we compared the results obtained by the RE system when using versus not using iSimp. The BioC XML format and schema described in the previous section were used to transfer the original data to iSimp and the RE system as well as to transfer the enhanced data from iSimp to the RE system. Besides adding and removing iSimp from the pipeline, no additional changes were made to the steps involved in the pipeline. This not only shows the interoperability of iSimp, but also proves that our proposed mechanism of using the BioC framework works as expected.

We tested this basic RE system on the BioNLP-ST 2011 GE task training corpus (14). Precision/Recall/F-value without simplification were 97.32/78.38/86.83 versus 97.42/81.62/88.82 with simplification. These results show that with the help of iSimp, the recall gap of 21.62 was reduced by 15% to 18.38, without introducing precision errors. In our previous and ongoing work (15), we have observed similar improvements in recall for various RE tasks.

In this exercise, we did not include agents because the GE corpora did not consider agents. But, because the above rules are most likely to be affected by noun phrase coordination, we believe simplification will benefit the agent extraction as well.

This exercise also illustrates the ability of sentence simplification to keep rules simple and yet achieve good results. Because patterns for simplification and RE are orthogonal, we do not need to multiply rules to consider all their combinations. An alternative way, as shown in the above example, is to treat sentence simplification as an independent task, and not for a particular RE. This way, we can focus on simple rules only. Sentence simplification is then applied to increase the recall of the original system.

### Simplification-annotated corpora in the BioC format

We provide a corpus marked with simplification constructs, using the BioC format (http://research.bioinfor matics.udel.edu/isimp/corpus.html). This corpus can be used by others to evaluate the performance of iSimp or other sentence simplifiers. The corpus consists of 130 Medline abstracts mentioning proteins and genes, with a total of 1199 sentences. The corpus contains three BioC files: (i) Medline abstracts of raw text, (ii) sentences that are split using the OpenNLP sentence detector and (iii) annotations of simplification constructs at the sentence level. Key files are also provided with additional information that describes the meaning of tags used in the BioC files and the annotation schema. The corpus uses the same DTD provided by BioC for validation.

Additionally, we have converted the BioNLP-ST 2011 GE corpus to the BioC format for our evaluation purposes, and this corpus can also be downloaded from the link given above.

### Conversion script

We provide a script to convert the BioNLP-ST corpus to the BioC format (https://bitbucket.org/udbiotmgroup/bionlp2bioc). The original text files (.txt) are split based on ‘newline’, and the various parts are stored into passage elements. Entities (in files.1) and event triggers (in files.2) are stored into appropriate passages based on their positions in the text files. Target annotations (in files.2), including events, relations, event modifications and equivalences, are recorded at the document level. If the annotation is marked by more than one continuous span of characters, the script creates several location elements. This also shows the generalizability of the BioC format, which allows multi-segmented annotations.

## Conclusion

In this study, we enhanced our sentence simplifier system, iSimp, to fully adopt the BioC format. We defined a unique BioC tag set for annotating simplification results and proposed a schema, which allows simplified sentences to be included in the BioC annotation file and be treated as part of the original collection. The proposed schema is different than the standard schema in that it can include words that are not part of the original text.

To illustrate the usefulness of iSimp with BioC, we examined its impact on a basic RE system. Evaluation on the BioNLP-ST 2011 GE task training corpus showed that, with sentence simplification provided by iSimp, the recall increased by 3.2%, which corresponds to a 15% reduction in recall error, without introducing precision errors. These corpora converted into the BioC format were made publically available together with the conversion script. Additionally, corpora we had previously developed for evaluating simplification performance of iSimp were made available in the BioC format, which may be used as public benchmarking corpora.

The corpora and the online demo of iSimp, using the BioC format, are available at http://research.bioinformatics.udel.edu/isimp/.

## Acknowledgements

The content is solely the responsibility of the authors and does not necessarily represent the official views of the National Institutes of Health. Any opinions, findings, and conclusions or recommendations expressed in this material are those of the author(s) and do not necessarily reflect the views of the National Science Foundation.

## Funding

Research reported in this article was supported by the National Library of Medicine of the National Institutes of Health under award number G08LM010720. This material is also based upon work supported by the National Science Foundation under Grant No. DBI-1062520. Funding for open access charge: National Science Foundation (DBI-1062520).

*Conflict of interest*. None declared.
